# COVID-19 Related Predisposition to Diabetic Ketoacidosis

**DOI:** 10.7759/cureus.36674

**Published:** 2023-03-25

**Authors:** Aadhithyaraman Santharaman, Kavin Raj, Kesavan Sankaramangalam, Sandesh Dewan, Saroj Sapkota, Sanya Chandna, Monarch Shah, Dhruv Singh, Mehak Bassi, Hongxiu Luo, Henry Redel

**Affiliations:** 1 Internal Medicine, Saint Peter’s University Hospital, New Brunswick, USA; 2 Internal Medicine, Rutgers Robert Wood Johnson Medical School/Saint Peter's University Hospital, New Brunswick, USA; 3 Infectious Disease, Saint Peter’s University Hospital, New Brunswick, USA

**Keywords:** cytokine storm syndrome, diabetes mellitus, hyperosmolar hyperglycemic syndrome, diabetic ketoacidosis, covid-19

## Abstract

Background and aims

Severe acute respiratory syndrome coronavirus 2 (SARS-CoV-2) can exacerbate hyperglycemia and can cause life-threatening diabetic ketoacidosis (DKA) in patients with diabetes mellitus (DM). The objective of this study is to compare the characteristics of diabetic COVID-19 patients with and without DKA and to determine the predictors of mortality in the setting of COVID-19 and DKA.

Methods

This is a retrospective single-center cohort study including patients admitted to our hospital with COVID-19 and DM from March 2020 to June 2020. Patients with DKA were filtered as per the diagnostic criteria set by the American Diabetes Association (ADA). Patients with hyperosmolar hyperglycemic state (HHS) were excluded. A retrospective analysis was performed, which included those who developed DKA and those with neither DKA nor HHS. The primary outcome measurement was mortality rate and predictors of mortality for DKA.

Results

Out of 301 patients with COVID-19 and DM, 30 (10%) had DKA and five (1.7%) had HHS. Mortality was significantly higher in the DKA group compared to the non-DKA/HHS group (36.6% vs 19.5%; OR: 2.38; p=0.03). After adjusting for parameters used for multivariate logistic model for mortality, DKA was no longer associated with mortality (OR: 2.08, p=0.35). The independent predictors for mortality were age, platelet count, serum creatinine, C-reactive protein, hypoxic respiratory failure, need for intubation, and need for vasopressors.

Conclusion

Our study demonstrates higher mortality rate in diabetic COVID-19 patients with DKA. Though direct and independent statistical association of mortality with DKA could not be proven in our multivariate logistic model, physicians must be vigilant in risk-stratifying and managing these patients in a timely manner.

## Introduction

The coronavirus disease 2019 (COVID-19) caused by the novel severe acute respiratory syndrome coronavirus 2 (SARS-CoV-2) has evoked a whole new perspective on the wide range of metabolic derangements caused by a viral infection. Within a span of three months since its genesis, the World Health Organization (WHO) declared this viral outbreak as a global pandemic [1]. It has been established that the presence of diabetes mellitus (DM) increases the probability of severe disease, admission to the intensive care unit, invasive ventilatory requirement, and mortality from COVID-19 [2,3]. A meta-analysis showed that patients with DM and COVID-19 are twice more likely to have a severe illness and twice more likely to die from it [4]. While there are adverse outcomes in those with pre-existing DM, there is also a possibility of new-onset DM triggered by this infection [5]. An early study in 2008 on patients affected by a related coronavirus, SARS-CoV, reported that hyperglycemia and a diagnosis of type 2 DM (T2DM) are independent predictors of mortality and morbidity [6]. One of the mechanisms by which SARS-CoV-2 can contribute to mortality is by inducing a state of hyperglycemic crisis with diabetic ketoacidosis (DKA) and hyperosmolar hyperglycemic state (HHS).

The hyperinflammatory states of both COVID-19 and DKA synergistically lead to the clinical downfall of such patients. A large retrospective study of 658 hospitalized patients with COVID-19 demonstrated that this infection can generate ketosis and ketoacidosis even in those without DM [7]. Studies have also shown that there has been an increasing incidence of DKA in emergency department encounters and hospital admissions that correlated with the peaks of the COVID waves [8,9]. In addition to having a longer hospital course, mortality is higher in patients with DKA when compared to those without. Mortality in COVID-19 patients with DKA has been reported to be around 50% [10-13], while mortality in DKA patients without COVID-19 is 0.67% [14]. This also correlated with higher rates of mechanical ventilation, renal replacement therapy, use of vasopressors, and use of glucocorticoids [15]. Clinical spectrum of endocrine derangements caused by SARS-CoV-2 also includes patients with combined DKA and HHS [16,17], and mortality is even higher in such cases [10,18]. In addition to the steroids used for the treatment of COVID-19, DKA and uncontrolled DM also are important risk factors for opportunistic infections such as mucormycosis [19]. The gravity of these complications invigorates the need to develop a better understanding of the relationship between COVID-19 and DKA.

We hypothesized worse clinical outcomes with an increased mortality in patients with DKA precipitated by SARS-CoV-2 infection. The objective of this study is to understand the characteristics of diabetic patients with COVID-19 and DKA and to determine the predictors of their mortality.

## Materials and methods

This is a retrospective single-center cohort study involving patients admitted during the first peak of COVID-19 from March 2020 to June 2020. Following approval from our IRB (study #20:56) and ethics committee, we identified a total of 301 subjects, aged 18 and above, in our electronic medical record system using the International Classification of Diseases 10th Revision (ICD-10) codes for the diagnosis of COVID-19 and DM. All of them were confirmed to have a positive nasopharyngeal RT-PCR for SARS-CoV-2. Data on basic demographic factors, medical history, laboratory values, hospital course including the modalities of treatment and final clinical outcome were manually extracted. All the laboratory values were from the day of admission, except for d-dimer, C-reactive protein (CRP), and lactate dehydrogenase (LDH), which were the highest recorded values during the hospitalization. We also obtained a list of diabetic patients who had positive blood cultures and/or respiratory viral panel during June 2018 to June 2020 using the ICD-10 codes.

We defined DKA cases as those who had serum glucose > 250 mg/dL, serum bicarbonate ≤ 18 mEq/L, anion gap > 10 mEq/L, and positive serum/urine ketones. We used the definition criteria set by the American Diabetes Association (ADA) [20]. Those with serum glucose > 600 mg/dL and serum bicarbonate > 18 mEq/L were considered to have HHS and were excluded from the analysis. Variables assessed include home medications, compliance, comorbidities, severity of COVID-19 infection, concurrent presence of sepsis or hypoxic respiratory failure, use of steroids during hospitalization, and mortality. A comparative analysis was performed between the DKA group and non-DKA/HHS group among COVID-19 diabetics.

Continuous variables are described as median and interquartile range except for age, which is described as mean and SD, as it was the only normally distributed variable. Categorical variables were described as counts and percentages. Wilcoxon rank-sum test was used for analyzing continuous variables except for age, for which the logistic regression was used with DKA as the outcome. Univariate and multivariate logistic regression models were used to analyze for mortality. A p-value of <0.05 was considered significant. All data were analyzed using the software Stata/SE Version 17 (StataCorp, College Station, TX).

## Results

Out of 301 patients with COVID-19 and DM, we found that 30 (10%) had DKA, of whom eight (26.7%) were diagnosed after admission. There were five (1.7%) with HHS who were excluded from analysis, leaving 266 patients in the non-DKA/HHS group.

In the comparative analysis performed between the DKA and non-DKA/HHS groups (Table 1), there was a male preponderance in the DKA subgroup (80% vs 57.5%, odds ratio [OR]: 2.95; 95% CI: 1.16-7.46; p=0.02). We also noticed that the BMI was significantly lower in the DKA group (28.3 vs 29.3; OR: 0.93; 95% CI: 0.86-0.;9, p=0.046). Patients with type 1 DM (T1DM) were significantly more likely to have DKA when compared to T2DM (OR: 12.91; 95% CI: 2.73-60.96; p<0.01).

**Table 1:** Univariate analysis between DKA and non-DKA/HHS DKA, diabetic ketoacidosis; DM, diabetes mellitus; DPP-4, dipeptidyl peptidase 4; GLP-1, glucagon-like peptide-1; HHS, hyperosmolar hyperglycemic state; CI, confidence interval; OR, odds ratio; SGLT2, sodium-glucose cotransporter-2; SIRS, systemic inflammatory response syndrome

Parameters	DKA (n = 30)	Non-DKA/HHS (n = 266)	OR (95% CI)	p-value
Age	57.9 ± 12.6	62.7 ± 15.1	0.97 (0.95-1.00)	0.09
Male	24 (80%)	153 (57.5%)	2.95 (1.16-7.46)	0.02
BMI	28.3 (24.1-29.7)	29.3 (25.6-34.1)	0.93 (0.86-0.99)	0.046
Medical history				
Newly diagnosed DM	3 (10%)	25 (9.4%)	1.07 (0.30-3.78)	0.91
Type 1 DM	4 (13.3%)	3 (1.13%)	12.91 (2.73-60.96)	< 0.01
Medication non-compliance	4 (13.3%)	7 (2.6%)	5.69 (1.56-220.7)	< 0.01
Home medication				
Insulin-dependent	16 (53.3%)	81 (30.4%)	2.61 (1.21-5.6)	0.01
Metformin	18 (60%)	152 (57.1%)	1.12 (0.52-2.42)	0.76
Sulfonylurea	8 (26.6%)	40 (15%)	2.05 (0.85-4.93)	0.1
SGLT2 inhibitor	3 (10%)	14 (5.2%)	2 (0.54-7.4)	0.29
GLP-1 receptor agonist	0 (0%)	8 (3%)		0.34
DPP-4 inhibitor	1 (3.3%)	40 (15%)	0.19 (0.02-1.47)	0.08
Thiazolidinediones	0 (0%)	6 (2.26%)		0.41
Meglitinides	2 (6.6%)	4 (1.5%)	4.67 (0.81-26.69)	0.06
Comorbidities				
Heart failure	0 (0%)	20 (7.5%)		0.12
Chronic obstructive pulmonary disease	1 (3.3%)	11 (4.1%)	0.79 (0.09-6.41)	0.83
Asthma	1 (3.3%)	9 (3.3%)	0.98 (0.12-8.05)	0.99
Chronic kidney disease	2 (6.6%)	54 (20.3%)	0.28 (0.06-1.21)	0.07
Malignancy	2 (6.6%)	25 (9.4%)	0.33 (0.04-2.54)	0.27
Labs				
White blood count	9.3 (6.5-14.7)	7.2 (5.6-9.6)	1.09 (1.03-1.17)	< 0.01
Hemoglobin	14.8 (12.7-15.9)	12.7 (11.3-13.9)	1.48 (1.20-1.81)	< 0.01
Platelet	299 (214-374)	223 (168-280)	1.00 (1.00-1.00)	< 0.01
Absolute lymphocyte count	0.95 (0.57-1.4)	0.99 (0.7-1.3)	0.99 (0.79-1.24)	0.95
C-reactive protein	162 (126-280)	134 (57-202)	1.00 (1.00-1.00)	0.02
Lactate dehydrogenase	463 (349-617)	319 (255-474)	1.00 (1.00-1.00)	< 0.01
D-dimer	624 (358-1524)	460 (270-1175)	1.00 (0.99-1.00)	0.49
Albumin	3.4 (3-3.8)	3.7 (3.3-3.9)	0.54 (0.27-1.06)	0.08
Urea	22 (16-44)	18 (11-30)	1.00 (0.99-1.02)	0.16
Creatinine	1.2 (0.9-1.5)	1 (0.75-1.5)	0.97 (0.81-1.16)	0.79
Alkaline phosphatase	97 (67-147)	82 (65-106)	1.00 (0.99-1.00)	0.4
Aspartate aminotransferase	34 (24-58)	37 (24-51)	1.00 (0.99-1.00)	0.34
Alanine transaminase	23 (17-38)	26 (17-47)	1.00 (0.99-1.00)	0.84
Lactate	2.3 (1.5-3.6)	1.2 (0.9-1.9)	0.93 (0.86-0.99)	0.04
Hemoglobin A1C	12.1 (10.4-14.1)	9.3 (7.6-12.4)	1.23 (1.02-1.50)	0.03
Hospital course				
≥2 SIRS upon presentation	20 (66.6%)	126 (47.3%)	2.22 (1.00-4.92)	0.04
ICU stay	23 (76.6%)	49 (18.4%)	14.55 (5.90-35.82)	<0.01
Hypoxic respiratory failure	19 (63.3%)	166 (62.4%)	1.04 (0.47-2.27)	0.92
Need for intubation	11 (36.6%)	38 (14.2%)	3.47 (1.53-7.87)	<0.01
Need for vasopressors	9 (30%)	25 (9.4%)	4.13 (1.70-9.98)	<0.01
Treatment				
Steroids	16 (53.3%)	68 (25.6%)	3.31 (1.53-7.13)	<0.01
Tocilizumab	8 (26.6%)	35 (13.1%)	2.4 (0.99-5.80)	0.047
Remdesivir	1 (3.3%)	8 (3%)	1.12 (0.13-9.20)	0.92
Convalescent plasma	5 (16.6%)	21 (7.8%)	2.33 (0.80-6.72)	0.11
Hydroxychloroquine	24 (80%)	176 (66.1%)	2 (0.80-5.18)	0.12
Azithromycin	17 (56.6%)	135 (50.7%)	1.26 (0.59-2.71)	0.54
Other antibiotics	24 (80%)	156 (58.6%)	2.82 (1.11-7.12)	0.02
Outcome				
Mortality	11 (36.6%)	52 (19.5%)	2.38 (1.06-5.31)	0.03

Patients with DKA were predominantly insulin-dependent (OR: 2.61; 95% CI: 1.21-5.6; p=0.01), medication non-compliant (OR: 5.69; 95% CI: 1.56-220.7; p<0.01), and poorly controlled DM (mean A1C 12.1% vs 9.3%, p=0.03). We did not see any statistically significant difference in the race and other comorbidities including congestive heart failure, chronic obstructive pulmonary disease, asthma, chronic kidney disease, and malignancy between both groups. There was also no statistically significant difference in other home medications between both groups, including SGLT2 (sodium-glucose cotransporter-2) inhibitors. Among the laboratory values, we found that the DKA subgroup had higher lactic acid (2.3 vs 1.2, p=0.04), CRP (162 vs 134, p=0.02), and LDH (463 vs 319, p<0.01).

Patients with DKA were significantly more likely to be septic with two or more systemic inflammatory response syndrome (SIRS) criteria satisfied upon presentation (OR: 2.22; 95% CI: 1.00-4.92; p=0.04) and to require ICU stay (OR: 14.55; 95% CI: 5.90-35.82; p<0.01), intubation (OR: 3.47; 95% CI: 1.53-7.87; p<0.01), and vasopressors (OR: 4.13; 95% CI: 1.70-9.98; p<0.01). A significantly higher proportion of patients with DKA received steroids (OR: 3.31; 95% CI: 1.53-7.13; p<0.01). Mortality was observed in 11 (36.6%) patients with DKA, which was significantly higher than those with neither DKA nor HHS (OR: 2.38; 95% CI: 1.06-5.31; p=0.03).

Using a multivariate logistic regression analysis (Table 2), we determined seven parameters that were independent predictors of mortality, namely, age, platelet count, serum creatinine, CRP, hypoxic respiratory failure, need for intubation, and need for vasopressors. After adjusting for all the parameters used for model, DKA was no longer associated with mortality (OR: 2.08; 95% CI: 0.44-9.76; p=0.35).

**Table 2:** Multivariate logistic model for mortality CI, confidence interval; DKA, diabetic ketoacidosis; SIRS, systemic inflammatory response syndrome

Parameters	Odds ratio	95% CI	P-value
Age	1.12	1.07-1.15	<0.01
Hemoglobin	0.97	0.79-1.19	0.8
Platelet	1.004	1.00-1.01	0.01
Creatinine	1.23	1.07-1.42	<0.01
C-reactive protein	1.01	1.00-1.01	<0.01
≥2 SIRS upon presentation	0.72	0.29-1.76	0.47
Need for vasopressors	26.16	5.39-127	<0.01
Need for intubation	3.72	1.22-11.35	0.02
Hypoxic respiratory failure	9.82	2.41-39.94	<0.01
Steroids	1.65	0.63-4.27	0.3
DKA	2.08	0.44-9.76	0.35

Out of a total of 286 patients with DM and positive blood cultures and/or respiratory viral panel during June 2018 to June 2020, 15 (5.2%) patients had developed DKA and two (0.7%) patients had developed HHS.

## Discussion

Our study corroborates the previously established data that diabetic patients with COVID-19 who develop DKA have a significantly higher mortality compared to those without DKA/HHS. However, it has not yet been established as an independent predictor of mortality. Even in our study, though DKA had significantly higher mortality, we could not establish it as an independent predictor of mortality. A recently published review article on this subject warrants the need for more studies with specific variables and larger sample size to establish a more definitive correlation [21]. While being unable to exclude the possibility of COVID-19 triggering new-onset DM, increased hospitalization of diabetic patients could explain the high rate of newly diagnosed DM in our study population. This could be related to acceleration of underlying pre-DM and it has been shown that patients with COVID-19 have a 40% increased risk of developing DM by the subsequent year [22]. Male patients, not necessarily obese, were found to be at risk for DKA in our study. The incidence of DKA in diabetic patients with COVID-19 is almost twice higher when compared to the incidence of DKA in diabetic patients with positive blood cultures and/or respiratory viral panel (10% vs 5.2%). Comparative analysis between these two groups could not be performed due to non-availability of data on all other variables. In order to curb the negative outcomes, it is prudent to understand the pathophysiology of DKA, which would help us in identifying at-risk population.

A state of acute insulin deficiency and profound insulin resistance in association with a surge of inflammatory response triggered by SARS-CoV-2 seems to be the primary mechanism by which these patients are predisposed to glycemic crisis [23]. This novel coronavirus finds its pathway into the human cells with its envelope spike glycoprotein binding to the ectoenzyme angiotensin-converting enzyme 2 (ACE2) [24]. Acute hyperglycemia has been shown to upregulate ACE2 expression, while chronic hyperglycemia downregulates it, making the cells vulnerable to inflammatory and damaging effects of the virus [25]. In addition to the heart, lungs, kidneys, and adipose tissue, ACE2 is also expressed in the pancreas [6,26,27]. Binding of SARS-CoV-2 to ACE2 can result in its downregulation, which leads to an unopposed action of angiotensin II from reduced conversion to angiotensin [28]. The resultant overactivation of the renin-angiotensin-aldosterone system (RAAS) leads to systemic and vascular insulin resistance through insulin-mediated Glut-4 translocation and impairment of endothelial nitric oxide production [29]. The action of angiotensin II on its receptors present on the exocrine, endocrine, and vascular cells of the pancreas can lead to vasoconstriction, decreased islet blood flow, and impairment of insulin release [30].

Yang et al. also suggested an acute damage of islets leading to insulin-dependent DM in patients with SARS [6]. There was a significantly higher proportion of patients with uncontrolled DM in our DKA subgroup. This is because in patients with uncontrolled hyperglycemia, “stunned” β-cells lead to an impairment in insulin secretion [31]. Acting as an environmental trigger, it is known that respiratory viral infections are associated with islet autoimmunity in patients with genetic predisposition to T1DM either through molecular mimicry or altered immune response [32]. A possibility of similar relationship between SARS-CoV-2 exposure and autoimmune DM should not be dismissed. In our study, 28 (9.3%) patients were newly diagnosed DM during admission for COVID-19 pneumonia.

SARS-CoV-2 can dysregulate the immune system and produce an inflammatory response characterized by high serum levels of pro-inflammatory cytokines (TNF-α, interleukin [IL]-1, and IL-6) and chemokines (IL-8) [33]. In severe cases, consumption of CD4+ and CD8+ T cells and a decrease of regulatory T cells produce an aggravated inflammatory response resulting in cytokine storm. Elevated levels of these proinflammatory cytokines have been associated with hyperglycemia, regardless of the patient’s diabetic status [34]. This inflammatory state set by COVID-19 can cause insulin resistance, which, in turn, increases oxidative stress in β-cells of pancreatic islets and peripheral tissues, resulting in impairment of insulin secretion [35]. Additionally, an increased production of counter-regulatory hormones during this state of hyperinflammation results in further suppression of β-cell function, impairment of glucose utilization in peripheral tissues, and increased hepatic and renal glucose production [36]. The pleotropic cytokine IL-6 has been linked to the pathogenesis of T1DM through activation of immune cells and alteration in homing of T cells to the sites of islet inflammation [37]. Elevated levels of IL-6 have been shown to be an early marker of sepsis in patients with DKA [38]. IL-6 has also been indicated in the facilitation of ketogenesis through induction of cellular insulin resistance in hepatocytes [39]. Mondal et al demonstrated IL-6 to be an independent predictor of DKA in patients with moderate-to-severe COVID-19 infection [40]. A rapid onset of this inflammatory process along with an upset in the glucagon/insulin ratio is the main precipitant of ketoacidosis in these patients. While it has not yet been established that COVID-19 causes DM, all of the above pathophysiologic effects point toward the possibility of the same.

Patients with DM and obesity are characterized by a chronic state of low-grade inflammation with defects in their innate and adaptive immunity [41]. Obese individuals also have an aggravated insulin resistance from physical inactivity. Surprisingly, in our study, patients in the DKA cohort had a lower BMI when compared to those without glycemic crisis (28.3 vs 29.3; OR: 0.93; 95% CI: 0.86-0.99; p=0.046). With regard to home medications, though majority of the patients in our study did not have a documented history of medication non-compliance, it was seen more significantly in the DKA group when present (OR: 5.69; 95% CI: 1.56-220.7; p<0.01). We must also harbor in mind the risk of euglycemic DKA seen in COVID-19 patients on SGLT2 inhibitors [42,43], though we did not find any similar association. Since the pharmacologic effects of SGLT2 inhibitors can persist for several days, patients can still develop euglycemic DKA even after discontinuation of the drug.

The gastrointestinal manifestations of COVID-19, such as anorexia, nausea, vomiting, and diarrhea, can contribute to volume depletion and increased lipolysis resulting in ketogenesis. These patients are admitted in a catabolic state, which is exacerbated by an inability to compensate for metabolic acidosis through renal and pulmonary mechanisms due to concurrent acute kidney injury (AKI) and impaired pulmonary gas exchange. Unopposed angiotensin II increases pulmonary vascular permeability and worsens the injury to lung parenchyma [44]. Fluid conservative management for patients with acute respiratory distress syndrome (ARDS) is counterintuitive to aggressive fluid replenishment for DKA, especially in the setting of septic shock. Matching the minute ventilation and maintaining an appropriate fluid balance is challenging in these patients. Moreover, there is a risk of hypokalemia while initiating intravenous insulin therapy due to the aldosterone-related renal potassium loss from RAAS overactivation. Subcutaneous rapid-acting insulin analogs should be considered for the treatment of mild-to-moderate DKA, especially outside of the ICU setting, to minimize the exposure of health care personnel to frequent fingerstick glucose checks and IV insulin drip titration [45].

While our study shows that patients with T1DM were significantly more likely to develop DKA, majority of patients in both cohorts had T2DM due to the worldwide higher relative prevalence. Development of AKI is a frequent complication in COVID-19 patients and is associated with an increased risk of adverse outcomes including mortality [46]. In our study, serum creatinine was determined to be an independent predictor of mortality (OR: 1.26; 95% CI: 1.09-1.46; p<0.01) as it is also a marker for organ dysfunction. We also noted a significantly higher rate of glucocorticoid and vasopressor use in patients with DKA, both of which can cause hyperglycemia and precipitate DKA. Steroids play a role in stress-related insulin resistance and can also delay the recovery of β-cell function [30]. Meticulous and regular titration of insulin dosage is warranted in these patients, especially while discharging them with steroids and insulin.

In patients with SARS-CoV infection, it was noted that more than 50% of them developed DM during hospitalization while not receiving any corticosteroids. Follow-up after three years showed that only 5% of these patients remained diabetic, indicating the transient nature of damage of islets by SARS-CoV infection [6]. Similar findings were seen in a short-term follow-up study in SARS-CoV-2 patients who developed acute-onset DM and DKA [47]. The requirement for exogenous insulin diminished significantly over four to six weeks, which could be related to the recovery of pancreatic β-cells following resolution of SARS-CoV-2 infection. These patients were able to resume their oral diabetic medications without insulin requirements during follow-up [48].

The limitation of our study was related to the retrospective nature of the study due to which causality could not be assigned. Some laboratory data were not available for all patients, which limited our ability to extend the statistical analysis to explore other associations and contributing factors for mortality. There is currently insufficient data to determine if SARS-CoV-2 poses an increased risk over other severe infectious diseases in predisposition to DKA. A collaborative prospective, multi-center approach would help in studying the long-term impact of COVID-19 on DM.

## Conclusions

In conclusion, physicians must be vigilant at all times to diagnose DKA with high degree of clinical suspicion in diabetic patients with COVID-19, especially in those with AKI, elevated CRP, hypoxic respiratory failure, need for intubation, and need for vasopressors. These factors should be considered while risk-stratifying diabetic patients to decrease worse clinical outcomes by a timely management. Insulin dosage should be carefully adjusted during follow-up due to the risk of hypoglycemia from tapering steroid doses and simultaneous recovery of pancreatic β-cell function.
